# Mitochondria: More than Just “Power Plants” in Stem Cells

**DOI:** 10.1155/2017/1826746

**Published:** 2017-10-12

**Authors:** Martin Stimpfel, Riikka H. Hämäläinen, Pascal May-Panloup

**Affiliations:** ^1^Reproductive Unit, Department of Obstetrics and Gynaecology, University Medical Centre Ljubljana, Ljubljana, Slovenia; ^2^A.I. Virtanen Institute for Molecular Sciences, University of Eastern Finland, Kuopio, Finland; ^3^Laboratoire de Biologie de la Reproduction, Centre Hospitalier Universitaire d'Angers, Angers, France

In mammalian cells, mitochondria are the only organelles containing DNA besides the nucleus. Mitochondrial DNA (mtDNA) comprises 37 genes of which 13 encode proteins and the others encode RNA molecules involved in the translation of these proteins. Although the number of proteins encoded by mtDNA is very small compared to that encoded by nuclear DNA, mtDNA and mitochondria are of vital importance for proper cellular function. In addition to their main function of producing ATP through oxidative phosphorylation, mitochondria play several additional vital roles as illustrated by the articles in this special issue. Since mitochondrial function and properties vary according to cell type, a better understanding of mitochondrial properties is of considerable interest particularly in the case of stem cells that may be used in cell therapy or regenerative medicine. Since many stem cell types rely mostly on a glycolytic metabolism rather than oxidative phosphorylation for energy production, they were long thought to be almost independent of mitochondrial function. However, recent advances have shown that proper mitochondrial function in stem cells is essential to maintain their self-renewal and differentiation abilities.

This special issue sums up recent findings concerning the essential role of mitochondria in stem cells. It includes selected reviews and an original article discussing the role and properties of mitochondria in stem cells not only from the perspective of basic science but also from the perspective of therapy.

When using stem cells for cell therapy, their heterogeneity may represent a challenge. One source for this cellular heterogeneity are mitochondria and as reviewed by D. C. Woods, different subpopulations of mitochondria can be present even within a single cell. This mitochondrial diversity and heterogeneity is believed to respond to cellular metabolic demands, but the underlying mechanisms are not yet completely understood. Since various specialized cells have specific metabolic demands and mitochondrial properties, D. C. Woods suggests that stem cells may serve as a useful model for elucidating the phenomenon of mitochondrial differentiation and the mechanisms leading to mitochondrial diversity and heterogeneity.

J. G. Lees et al. focus on pluripotent stem cells and on the precise role played by mitochondrial metabolites in maintaining pluripotency. The authors discuss the role of mitochondria in the epigenetic modifications on chromatin and highlight the impact of hydrogen peroxide, an important by-product of mitochondrial respiration. In high concentration, hydrogen peroxide may stimulate proliferation of pluripotent stem cells via hypoxia-inducible factor *α* (HIF*α*) signaling, but this process is dependent on physiological oxygen level. Oxygen is therefore recognized as one of the key factors influencing the metabolism and behavior of pluripotent stem cells and has even been proposed to be used as a selective factor.

The mitochondria of specialized somatic cells differ substantially from those of pluripotent stem cells. Thus, when somatic cells are reprogrammed to the pluripotent state, the mitochondria undergo remodeling through mitochondrial fission and mitophagy. Several studies show that these processes are critically involved in nuclear reprogramming. J. Prieto and J. Torres review these findings in normal cells and linked them with development of human malignancies.

Interestingly, mitochondria can be transferred between cells. The mechanisms of this phenomenon and its potential therapeutic applications are reviewed here in two articles by M.-L. Vignais et al. and A. Caicedo et al. Mitochondrial transfer has several physiological functions. It serves mainly in rescue operations with healthy cells donating mitochondria to damaged ones. Moreover, recent data have shown that mitochondrial transfer is also involved in mitochondrial degradation through transcellular mitophagy. Thus, it constitutes crucial mechanism for maintaining mitochondrial homeostasis in multicellular organisms. Natural mitochondrial transfer may occur through intracellular connections such as tunneling nanotubes or by the secretion of cellular bodies as in the case of microvesicles. Artificial mitochondrial transfer involves also other mechanisms such as the injection of mitochondria in recipient cells, the coincubation of mitochondria with recipient cells, or the use of various chemical compounds. With stem cell therapy, artificial mitochondrial transfer has been proposed for the treatment of mitochondrial retinopathies, muscular skeletal syndromes, and other mitochondrial diseases. However, since mitochondria contain DNA, such treatments raise ethical and legal questions that will need to be resolved before the technique comes into general use.

D. Yu et al. studied the methylation of mtDNA in human fetal heart mesenchymal stem cells (MSCs) during the process of senescence induced by chronic exposure to oxidative stress and low serum environment. The authors were able to identify for the first time the specific regions of mtDNA that were hypomethylated upon senescence. More precisely, COX1, which encodes a subunit of the cytochrome c oxidase complex, the main enzyme involved in mitochondrial oxidative phosphorylation, was hypomethylated and consequently upregulated. COX1 upregulation was also induced by knockdown of methyltransferases DNMT1, DNMT3a, and DNMT3b. However, the precise role of the upregulation of COX1 in senescence remains to be elucidated. The authors suggest that the hypomethylation of specific mtDNA regions could be used as a biomarker of the senescence of MSCs.

In summary, this special issue offers an overview of the major findings concerning the properties and the physiological roles of mitochondria in stem cells. These results should lead to new scientific insights into mitochondrial function in the context of potential therapeutic applications in the future.

*Martin Stimpfel* *Riikka H. Hämäläinen* *Pascal May-Panloup*

